# Supplementary material for: On-chip single-crystal plasmonic optoelectronics for efficient hot carrier collection and photovoltage detection
Source: Light Sci Appl. 2025 Sep 16;14:325. doi: 10.1038/s41377-025-02030-6 (PMC12441122; doi:10.1038/s41377-025-02030-6)
Supplement: Supplementary file 1 — Supplementary Materials for On-chip Single-crystal Plasmonic Optoelectronics for Efficient Hot Carrier Collection and Photovoltage Detection [file 41377_2025_2030_MOESM1_ESM.docx]

**Supplementary Materials for**

**On-chip Single-crystal Plasmonic Optoelectronics for Efficient Hot Carrier Collection and Photovoltage Detection**

*Yunxuan Zhu^1,2,*^, Sai. C. Yelishala^1^, Shusen Liao^2^, Jackson* *Shropshire^3^, Douglas Natelson^2,4,5,*^ and Longji Cui^1,6,*^*

^1^Paul M. Rady Department of Mechanical Engineering, University of Colorado Boulder, Boulder, CO 80309, United States.

^2^Department of Physics and Astronomy, Rice University, Houston, TX 77005, United States.

^3^Department of Chemical and Biological Engineering, University of Colorado Boulder, Boulder, CO 80309, United States.

^4^Department of Electrical and Computer Engineering, Rice University, Houston, TX 77005, United States.

^5^Department of Materials Science and Nanoengineering, Rice University, Houston, TX 77005, United States.

^6^Materials Science and Engineering Program and Center for Experiments on Quantum Materials, University of Colorado Boulder, Boulder, CO 80309, United States.

Email addresses: [yunxuan.zhu@colorado.edu](mailto:yunxuan.zhu@colorado.edu), [sai.yelishala@colorado.edu](mailto:sai.yelishala@colorado.edu), [sl147@rice.edu](mailto:sl147@rice.edu), [jackson.shropshire@colorado.edu](mailto:jackson.shropshire@colorado.edu), [natelson@rice.edu](mailto:natelson@rice.edu), and [longji.cui@colorado.edu](mailto:longji.cui@colorado.edu)

^*^Corresponding authors: [yunxuan.zhu@colorado.edu](mailto:yunxuan.zhu@colorado.edu) (Y.Z), [natelson@rice.edu](mailto:natelson@rice.edu) (D.N), [longji.cui@colorado.edu](mailto:longji.cui@colorado.edu) (L.C)

**1. A simplified model of carrier temperatures**

A full theoretical model of the nanogap under illumination and/or bias is extremely challenging, requiring a treatment of the local plasmonic excitations, the spatially and temporally dependent electronic distribution, and the open nature of the system, including diffusion of the carriers and the energy content. Inferring an open-circuit photovoltage would further require some definition of the asymmetry of the junction (either in the generation of hot carriers or the tunneling kinetics) that leads to a net hot-carrier tunneling current under illumination. Such a model is well beyond the scope of the present work. However, some insight can be gained by considering a much-simplified treatment, adapted from approaches applied to nanoparticles, that treats the energy density, effective temperature of a population of locally thermalized electrons, and the lattice temperature as *local* variables.

After the electrical or optical excitation of the hot carriers, within the first tens to hundreds of femtoseconds, excited nonthermalized hot electrons undergo rapid electron-electron scattering, quickly relaxing their energy and establishing a steady-state hot carrier distribution characterized by a high effective temperature. Electron-phonon scattering subsequently comes into play to further relax the hot carrier energy to the lattice on the picoseconds time scale. The whole process in this simplified system can be well described by three coupled differential equations(*1*),

$$\frac{\partial N\left( t \right)}{\partial t}=-\gamma_{e-e}N\left( t \right)-\frac{G_{e-ph}}{C_{e}}N\left( t \right)+P_{ex}\left( t \right) (1)$$

$$C_{e}\frac{\partial T_{eff}\left( t \right)}{\partial t}=-G_{e-ph}\left( T_{eff}\left( t \right)-T_{l}\left( t \right) \right)+\gamma_{e-e}N\left( t \right) (2)$$

$$C_{l}\frac{\partial T_{l}(t)}{\partial t}=G_{e-ph}\left( T_{eff}\left( t \right)-T_{l}\left( t \right) \right)+\frac{G_{e-ph}}{C_{e}}N\left( t \right)-P_{sub}\left( t \right) (3)$$

where $N\left( t \right)$ is the energy density of the excited non-thermal electrons, $\gamma_{e-e}$ is the electron-electron scattering rate, $G_{e-ph}$ is the electron phonon coupling constant. $C_{e}, T_{eff}\left( t \right)$ and $C_{l}, T_{l}\left( t \right)$ are the heat capacity and temperature (effective temperature for steady state hot carriers) of electron and phonon respectively. $P_{ex}\left( t \right)$ and $P_{sub}\left( t \right)$ are the absorbed optical excitation power and energy relaxation rate to the external environment (substrate).

Augmenting this local model to account at least a bit for sample geometry, different from the previous study on large monocrystalline gold micro flakes, the bow-tie shaped constriction employed here will introduce more boundary scattering for the electrons and thus providing an additional relaxation channel that needs to be considered. Following the paper by Tomchuk and Fedorovich(*2*, *3*), the energy transferred to the lattice can be expressed by,

$$P_{e-ph}=\Gamma\left( T_{eff}^{2}-T_{l}^{2} \right) (4)$$

Where $\Gamma$ is a factor that contains the characteristic length $L$ of the constriction area,

$$\Gamma=\frac{\pi^{2}}{4}\left( \frac{m^{2}k_{B}^{2}}{\hbar^{3}M} \right)E_{F}\frac{1}{L} (5)$$

here $m$ and $M$ are the mass for electron and atom, $E_{F}$ is the Fermi energy, $k_{B}$ is the Boltzmann constant, and $\hbar$ is the reduced Planck’s constant. Eq. (4) is then used to substitute for the electron phonon energy relaxation term in the bulk $G_{e-ph}\left( T_{eff}\left( t \right)-T_{l}\left( t \right) \right)$ in Eq. (5).

Under CW optical excitation, the system will eventually reach a steady state (the time-dependent terms in Eq. (1-3) all vanish), where the relative electron effective temperature difference can be compared by inserting the physical parameters into Eq. (4). According to previous studies(*1*, *4*), $G_{e-ph}$ are taken as $2.2\times{10}^{16} Wm^{-3}K$ and $2.0\times{10}^{16} Wm^{-3}K$ for SC and PC gold respectively. $C_{e}$ is $67 Jm^{-3}K^{-2}$ and $E_{F}$ is 5.52 eV for gold. Assuming one side is unsymmetrically illuminated for the optimal OCPV signal and a beam spot size of 2um, $L$ is taken to be 700 nm. Electron-electron scattering rate is taken as 18 THz and 24 THz for SC and PC from previous ultrafast pump-probe experiments(*1*). The relative effective temperature difference for SC and PC gold can then be estimated to be less than 6%, which is consistent with the extracted effective temperature value from the EL data. Thus, this simplified model is consistent with the experimental observation that effective temperatures in the steady state of SC and PC junctions are quite similar. As stated in the main text, this implies that the tunneling kinetics of hot carriers in the SC and PC junctions, explicitly neglected here, must be the origin of the large difference in OCPV between the SC and PC junctions.

**2. Finite element simulation**

A finite element method (FEM) simulation is performed to model the electromagnetic response of a gold nanowire system using COMSOL Multiphysics with the electromagnetic wave, frequency domain (ewfd) module. The whole system for modelling includes the gold nanowire (single crystal gold and evaporated gold), 2 $\mu$m thick SiO_2_ substrate on Si and the vacuum above the nanowire. The values of dielectric function of single crystal gold and evaporated gold are obtained from the previous studies^55^. The material properties of the SiO_2_ and Si are selected in the COMSOL material library. The whole geometry is surrounded by a perfect match layer (PML) to mimic an open and nonreflecting infinite domain. The laser is set to be a perfect Gaussian beam with the spot size to be 2 $\mu$m in radius used in the experiment^52^. The polarization angle of the laser is set to be either parallel (0 degree) or transverse (90 degree) to the nanowire. A minimum mesh size of 0.5 nm was selected to ensure numerical accuracy for resolving small gaps and interfaces, with at least two mesh elements being covered inside the gap. We first calculate the background field in the free space, and then we use the results to calculate the full scattered field to obtain the enhancement shown in the main text.


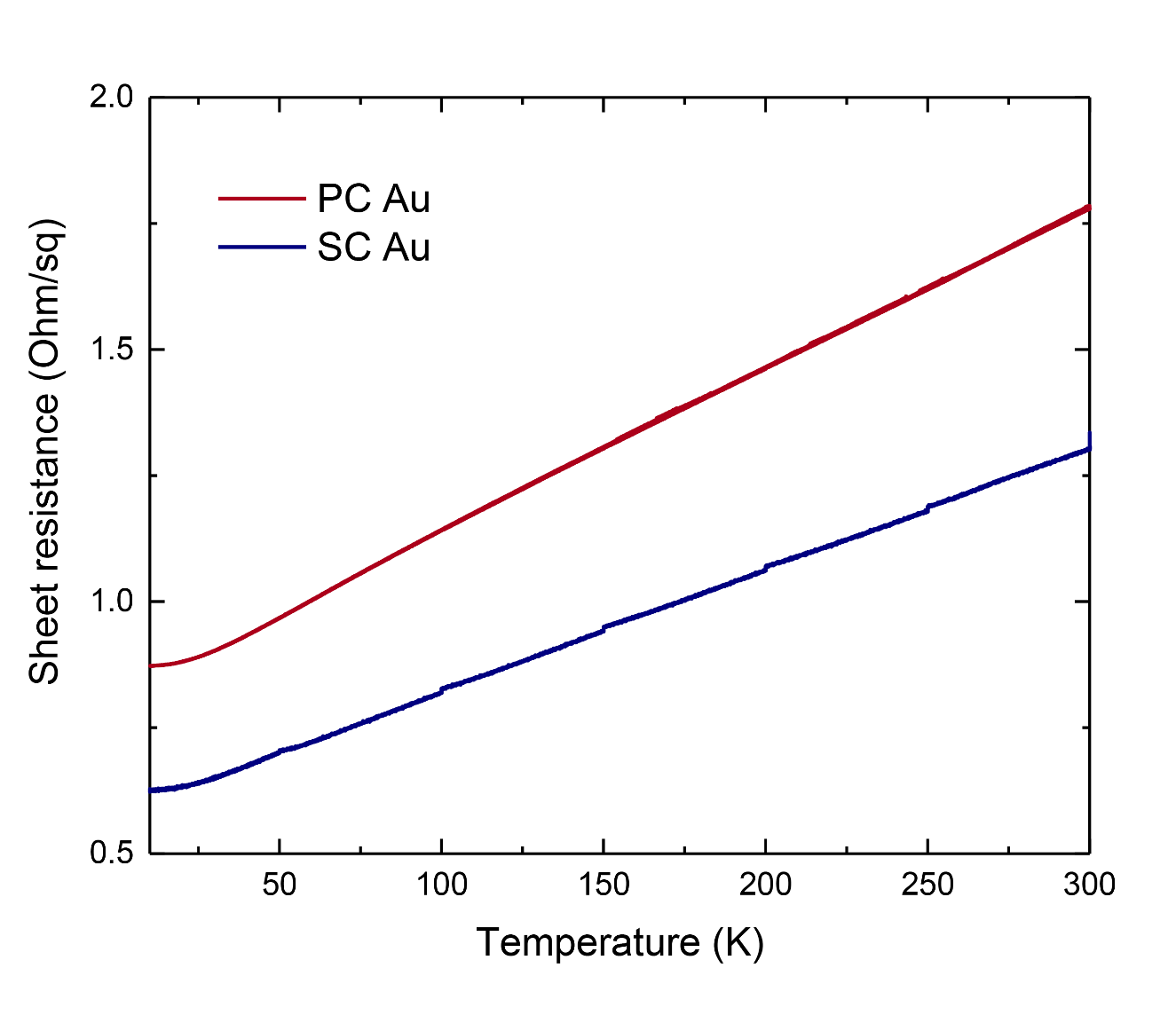


Fig. S1. Sheet resistance of 20nm thick gold as a function of temperature for PC and SC Au respectively. The geometry for PC Au nanowire is 100 μm long, 2 um wide. The geometry for SC Au nanowire is 1.8 μm long, 280 nm wide.


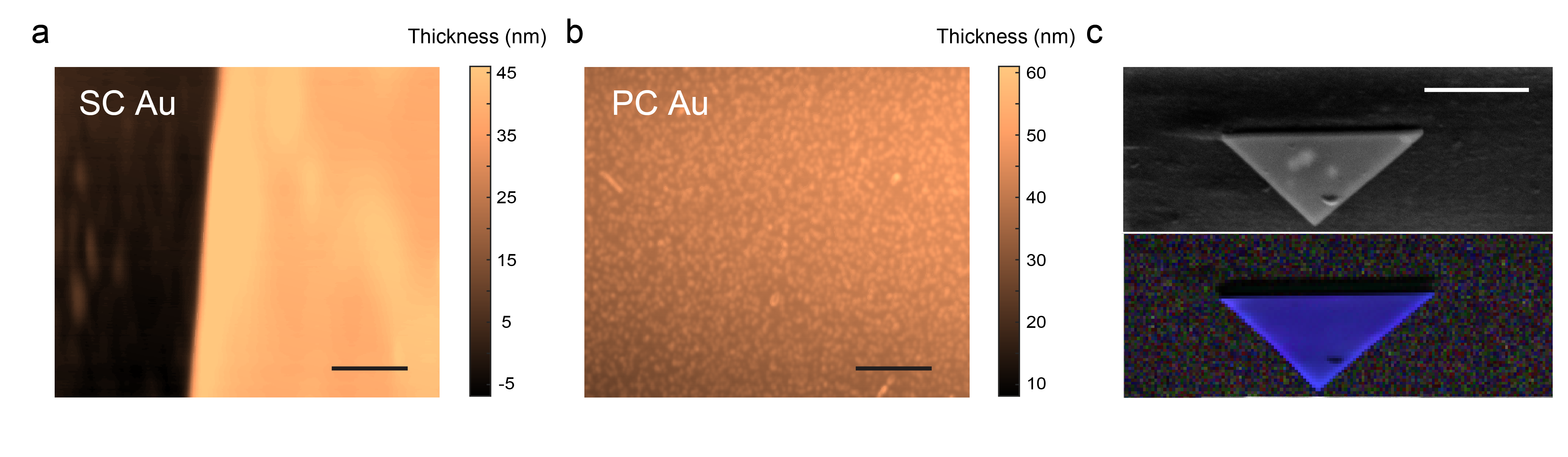


Fig. S2. (a-b) AFM scanning image for SC and PC Au respectively. The range of the color bar have been chosen to be the same for fair comparison. The scale bar in the figure is 2 um. (c) SEM image (top) and EBSD inverse pole figure (IPF) map (bottom) showing the crystallographic orientation of a single crystalline Au flake deposited on top of an e-beam evaporated polycrystalline Au film. The triangular region exhibits a uniform color, indicating a consistent crystallographic orientation corresponding to the Au (111) plane. In contrast, the surrounding evaporated Au shows multiple grain orientations and visible grain boundaries. The scale bar in the figure is 2 um.

**References**

1. C. O. Karaman, A. Y. Bykov, F. Kiani, G. Tagliabue, A. V. Zayats, Ultrafast hot-carrier dynamics in ultrathin monocrystalline gold. *Nature Communications 2024 15:1* **15**, 1–8 (2024).

2. R. D. Fedorovich, A. G. Naumovets, P. M. Tomchuk, Electron and light emission from island metal films and generation of hot electrons in nanoparticles. *Phys Rep* **328**, 73–179 (2000).

3. M. Buret, A. V. Uskov, J. Dellinger, N. Cazier, M.-M. Mennemanteuil, J. Berthelot, I. V. Smetanin, I. E. Protsenko, G. Colas-des-Francs, A. Bouhelier, Spontaneous Hot-Electron Light Emission from Electron-Fed Optical Antennas. *Nano Lett* **15**, 5811–5818 (2015).

4. A. M. Dowgiallo, K. L. Knappenberger, Ultrafast electron–phonon coupling in hollow gold nanospheres. *Physical Chemistry Chemical Physics* **13**, 21585–21592 (2011).
